# Measuring trust: a text analysis approach to compare, contrast, and select trust questionnaires

**DOI:** 10.3389/fpsyg.2023.1192020

**Published:** 2023-11-15

**Authors:** Areen Alsaid, Mengyao Li, Erin K. Chiou, John D. Lee

**Affiliations:** ^1^Department of Industrial and Manufacturing Systems Engineering, University of Michigan-Dearborn, Dearborn, MI, United States; ^2^Department of Industrial and Systems Engineering, University of Wisconsin-Madison, Madison, WI, United States; ^3^Department of Human Systems Engineering, Arizona State University, Mesa, AZ, United States

**Keywords:** trust, trust assessment, trust measurement, questionnaires, text analysis, trust layers

## Abstract

**Introduction:**

Trust has emerged as a prevalent construct to describe relationships between people and between people and technology in myriad domains. Across disciplines, researchers have relied on many different questionnaires to measure trust. The degree to which these questionnaires differ has not been systematically explored. In this paper, we use a word-embedding text analysis technique to identify the differences and common themes across the most used trust questionnaires and provide guidelines for questionnaire selection.

**Methods:**

A review was conducted to identify the existing trust questionnaires. In total, we included 46 trust questionnaires from three main domains (i.e., Automation, Humans, and E-commerce) with a total of 626 items measuring different trust layers (i.e., Dispositional, Learned, and Situational). Next, we encoded the words within each questionnaire using GloVe word embeddings and computed the embedding for each questionnaire item, and for each questionnaire. We reduced the dimensionality of the resulting dataset using UMAP to visualize these embeddings in scatterplots and implemented the visualization in a web app for interactive exploration of the questionnaires (https://areen.shinyapps.io/Trust_explorer/).

**Results:**

At the word level, the semantic space serves to produce a lexicon of trust-related words. At the item and questionnaire level, the analysis provided recommendation on questionnaire selection based on the dispersion of questionnaires’ items and at the domain and layer composition of each questionnaire. Along with the web app, the results help explore the semantic space of trust questionnaires and guide the questionnaire selection process.

**Discussion:**

The results provide a novel means to compare and select trust questionnaires and to glean insights about trust from spoken dialog or written comments.

## 1. Introduction

Trust has been studied in myriad contexts, from the internet to consumer products, healthcare, the military, and transportation. One challenge for advancing trust research is being able to measure trust precisely, and in a way that can generalize across contexts.

The study of trust in diverse contexts has resulted in multiple definitions: as a belief, an attitude, an intention, and a behavior. While these definitions are conceptually distinct, they are also interrelated; beliefs are derived from an individual’s past experiences, affective processing of beliefs governs attitudes, attitudes modulate intentions, and intentions are turned into behaviors (Ajzen and Fishbein, 1980). Nonetheless, trust is fundamentally an attitude. In the study of trust for systems design, trust is considered as a mediator between beliefs and behaviors, and hence, trust is defined as “the *attitude* that an agent will help achieve an individual’s goals in a situation characterized by uncertainty and vulnerability” (Lee and See, 2004). Because of the variation in how trust has been conceptualized and subsequently operationalized, there exists a multitude of trust measures (Kohn et al., 2021).

Observable behaviors have been used as proxy measures of trust because they are often seen as more objective and less obtrusive than self-report measures. Behaviors are also often the main outcomes of interest when it comes to the study of trust, such as understanding what affects people’s decisions to rely on the advice of a virtual real estate agent (Cassell and Bickmore, 2000), with trust being one important factor. Some behavioral indicators used to study trust have included: compliance, reliance, eye gaze, voice, and facial expression, and even pedal presses in automated vehicles (Meyer and Lee, 2013; Price et al., 2017; Lee et al., 2019).

Behavioral measures of trust are useful for understanding trust as a socio-cognitive construct between agents interacting in real-time (Takayama, 2009; Schilbach et al., 2013) and can serve as inputs to models used to dynamically predict human behavior in specific task contexts (Domeyer et al., 2018; Yang et al., 2021). However, behavioral measures are often tied to a specific context or experimental setup and are considered indirect measures of trust because it is possible to engage in trust-related behaviors without actually involving trust (Chiou and Lee, 2021). Therefore, when generalizing from laboratory studies to real-world scenarios, there is a substantial risk of misinterpreting or misapplying behavioral measures of trust. This risk is often associated with the potential for compromised construct validity, because of the lack of ground truth, which hinders the progress in establishing a robust theoretical foundation of trust (Campbell and Fiske, 1959; Lee and See, 2004).

In contrast to behavioral measures, questionnaires (i.e., self-report) are a more direct measures of trust, because trust is fundamentally an *attitude* and not a behavior. Therefore, asking a person about their attitude and closely associated factors, such as their beliefs and expectations, is important for understanding that person’s trust. Although a person’s reflective responses are also imperfect measures and not without limitations, questionnaires have the added advantage of being straightforward to administer, are typically rigorously developed based on trust theory, have established methods for validating empirically, and can more easily generalize across task contexts. Indeed, questionnaires have been widely used to measure trust. Yet, the literature indicates that several trust questionnaires have been developed for more specific task environments, perhaps to increase the sensitivity of the instrument. This has led to many trust questionnaires spanning multiple fields and contexts (Kohn et al., 2021).

The large pool of existing questionnaires presents a challenge to researchers in selecting the appropriate questionnaire. Questionnaire selection depends on several factors such as the application domain, the context, and the trust layer of interest. Questionnaire items can characterize different layers of trust such as dispositional, learned, and situational trust (Hoff and Bashir, 2014). A comparison between the questionnaires and their constituent items and words can guide the selection process. However, these relationships have not been systematically explored.

A recent paper described how nine questionnaires, measuring trust in automation specifically, related to one other based on a semantic network analysis of their constituent words (Jeong et al., 2018). Using Latent Semantic Analysis (LSA) in combination with network analysis, the paper identified 14 highly central words that could be used to create an integrated scale. While promising, this paper focused on the similarity between keywords. Focusing on the words only might overlook the contextual information contained by a questionnaire item or a questionnaire as a whole. In this study, we investigate the similarity across the words, the questionnaire items, and the questionnaires.

Text analysis could be used to reveal connections between the many different trust questionnaires. These connections can be condensed and visualized in two-dimensional semantic spaces. The manifestation of these connections in the semantic space at different levels of analysis (i.e., words, items, and questionnaires) allows researchers to compare and select the questionnaires that best support their research needs. Hence, text analysis provides one lens for considering the differences and similarities between various trust questionnaires.

Accordingly, the present analysis is not aimed at developing a new scale, nor finding a single ideal one, as there is no single ideal questionnaire that works for all experiment and contexts (Kohn et al., 2021). However, it gathers the most commonly used questionnaires in three most common research area: humans, e-commerce, automation. It also provides high-level comparison and guidance to researchers to choose the best-suited questionnaire for their research question.

### 1.1. A primer on text analysis

In text analysis, words are often represented as embeddings. Embeddings are vectors of numbers that describe the location of a word in a high-dimensional semantic space relative to other words (Pennington et al., 2014). For example, words like “cat” and “dog” would be closer to each other than “cat” and “mailbox.” Words with similar meanings have similar vector representations and will thus be close to each other in the semantic space. The vector representation of words allows for mathematical operations that quantify the similarities of words and hence allows for advances in natural language processing applications like sentiment analysis and text autocompletion.

Methods that learn the vector representation of words are categorized into (i) global matrix factorization methods and (ii) local context window methods (Pennington et al., 2014). The first method exploits statistical information contained by the words, such as Latent Semantic Analysis (LSA). The intuition is to extract relationships between the words in the corpus, assuming that words similar in meaning will appear in similar contexts (Landauer and Dumais, 2008). LSA relies on the frequency of word occurrence and ignores the context in which the words appear. It represents the text data in a corpus matrix that consists of word frequencies in each document. Each word occurrence in each document is counted, and the entire matrix is reduced using Singular Value Decomposition (SVD). As a result, documents that share more words are considered similar, even if the similar words were used in a different context [e.g., the “bank” in “river bank” and “bank ATM” is considered equal (Hu et al., 2016)]. LSA produces semantic spaces that are high-level abstractions that are useful but lack context information.

The second method uses skip-gram models to capture the local context in which the word occurs. In skip-gram models, a constant length window is moved along the corpus, and a neural network is trained to capture the co-occurrence of words in that entire window, and to predict context based on the central word (Altszyler et al., 2016). One example is a technique called word2Vec (Mikolov et al., 2013), which preserves the local context and provides a more precise description of the relationships between words compared to LSA and SVD. In word2Vec, embeddings are estimated by predicting words based on the words in the predefined window which enables the embeddings to capture relationships between words such that vector operations on the embeddings can complete word analogies in a meaningful fashion. In line with this paper’s goals, we chose Global Vectors for Word Representation (GloVe) because it is a suitable approach for text analysis tasks that require considering the context within the data and the broader context of spoken language (Pennington et al., 2014). GloVe combines the benefits of global factorization and local context methods: it uses the statistical information contained by the words while also accounting for context by considering the co-occurrence statistics of words within a corpus. GloVe is trained on the non-zero elements of aggregated global word-to-word co-occurrence probability matrix and shows improved interpretability and accuracy compared to Word2Vec.

The vector representation of the words defines the position of each word in a high dimensional space, typically 100–500 dimensions. However, high-dimensional data is hard to visualize making it hard to identify what words similar to each other (Patel, 2016). Dimensionality reduction techniques reveal the underlying structure of the data. Principal component analysis (PCA) is a common dimensionality reduction technique that finds the linear combinations of the variables that capture the most variance in a dataset (Hubert et al., 2005). t-Distributed Stochastic Neighbor Embedding (t-SNE) is another technique that accommodates non-linear relationships between the variables and more precisely captures the micro-structure of the data: it maps similar instances to nearby points and dissimilar instances to distant point in the lower-dimensional space (van der Maaten and Hinton, 2008).

In this paper, we use a non-linear dimensionality reduction technique, called Uniform Manifold Approximation and Projection (UMAP), which captures non-linear relationships, like t-SNE, but in a more reproducible and computationally efficient manner (McInnes et al., 2018). UMAP creates a low-dimensional representation where similar items are near each other, and it preserves the micro and macro structure of the data, which is appropriate for this study since it is beneficial to understand both the inter-cluster and intra-cluster relationships for the trust questionnaires, their constituent items, and words (i.e., highlighting how they relate or diverge). For visualization purposes, we used a two-dimensional space. However, the results of dimensionality reduction might not be directly comprehensible since it is highly non-linear (McInnes et al., 2018), nonetheless, they can reveal important relationships between the variables (Alsaid et al., 2018; Alsaid and Lee, 2022). For more details on text analysis and dimensionality reduction techniques, see Appendix A.

### 1.2. Research objective

In this paper, we use word-embedding text analysis to understand different aspects of trust questionnaires and selecting the appropriate ones. First, we conduct a scoping literature review to gather existing questionnaires. Second, we apply text analysis techniques to quantify the relationships between the words used in the questionnaires, the questionnaire items within the questionnaires, and the overall questionnaires. These relationships were quantified using GloVe vector representations of the words. Third, we develop charts that quantify the composition of each questionnaire (i.e., application domain and trust layer composition) to guide researchers to select a questionnaire suited for the research task at hand. Finally, we generate a lexicon of the trust-related words that could be used to develop trust questionnaires and trust-focused sentiment analysis. The results are implemented in a web application that can help the researchers compare and contrast the different trust questionnaires and select the best fit for their research needs.

## 2. Method

### 2.1. Compiling and labeling the corpus

A scoping literature review (Grant and Booth, 2009) using google scholar was conducted using the keywords: “trust in automation, trust in humans, trust in e-commerce, trust, assessment, scales,” their variants (e.g., “technology,” “robots,” “interpersonal trust,” “surveys,” “questionnaires”) and their combinations.

Titles and abstracts were read to select those that developed or used rating-based trust measures. Then all articles were read in detail, and only *unique* developed questionnaires were included in the final selection and multiple questionnaires had overlapping items. A total of 80 articles were downloaded that met these inclusion criteria. Of these 80 articles, 46 questionnaires were extracted, with a total of 626 questionnaire items. After assessing the final selection of questionnaires, the questionnaires were categorized and labeled based on the domain for which they were developed (Chita-Tegmark et al., 2021):

1. Automation: questionnaires developed for assessing trust in automation, including robots and technology more generally.
2. E-Commerce: questionnaires developed to assess consumers’ trust in brands, trust in retailers’ websites, and online shopping in general.
3. Human: questionnaires developed to assess interpersonal trust.

Because not all questionnaire items assessed the same layer of trust, the items within each questionnaire were also categorized and labeled according to the layer of trust that they measured, based on Hoff and Bashir’s model of trust layers (2014), for its comprehensiveness:

1. Dispositional: measures a person’s general tendency to trust, independent of context or a specific system. Dispositional trust arises from long-term biological and environmental influences.
2. Learned: measures a person’s trust based on previous experiences with a specific automated system.
3. Situational: measures trust in a specific context or situation including both the external environment and the internal, context-dependent characteristics of the operator.

Earlier trust scales primarily examined interpersonal trust and dispositional trust as measures of individual differences and personal characteristics. However, as technology advanced, there emerged a growing interest in studying trust within specific domains such as e-commerce and automation. Furthermore, the scales appeared to span a spectrum ranging from general assessments, such as Rotter (1967) scale, which assessed overall trusting tendencies, to more specific questionnaires like Körber (2018) which focuses on particular aspects of automation.

### 2.2. Data cleaning

Once the corpus was compiled and labeled, the first step of our text analysis was to pre-process the data. We converted all words to lowercase, removed one-letter words, and punctuation. We also converted plural words to their singular form, such as ‘decisions’ to ‘decision. To focus the analysis on words relevant to trust assessment, we excluded stop words. Stop words refer to unimportant, uninformative, frequently used words such as pronouns, prepositions, and auxiliary verbs. Here, we used a list of stop words from the tidytext package, specifically, the Onix stop word lexicon. The Onix stop word list was moderately aggressive in removing words compared to other stop word lists. The Onix list includes words such as “become,” “know,” “fully,” “great” and “interesting.” In addition, we removed words that referred to either the trustor or trustee like “product,” “system,” “user,” “technology,” or “consumer” because of their high frequency and limited relevance to making conceptual distinctions regarding trust. A complete list of removed word can be found in Appendix C.

### 2.3. Data analysis

Using the Wikipedia 2014 + Gigaword 5 pre-trained word vectors dataset provided on the GloVe website (Pennington et al., 2014), we calculated embeddings for each word, questionnaire item, and questionnaire. At the word level, we matched the words’ vector embeddings with those in the pre-trained data. At the item level, we calculated the log odds ratios weighted by an uninformative Dirichlet prior for the words in each item. Using the log odds increases the weights for words that are common in a specific item, and relatively uncommon among all other items. This method gives greater weight to distinguishing words (Monroe et al., 2008). The log odds ratios were then used to create a weighted mean of the embeddings of the words that comprise each item. We used this same process to calculate an embedding for each questionnaire. For more details on the log odds ratio calculations see Appendix A.

To develop the trust lexicon, we calculated the log odds ratio of the words in the trust questionnaires, given the 5,000 most common English words list from the wordfrequency website (Davies and Gardner, 2010), and extracted the 20 most unique trust words. We calculated the cosine similarity distance between each of the 20 words and the GloVe word embeddings, similar to the approach of Fast et al. (2017), and for each of those word, we extracted the five closest words.

### 2.4. Software tools

We used R statistical software (R Development Core Team, 2016) to create plots with the ‘ggplot2’ package (Wickham and Winston, 2019); for data cleaning, we used the ‘tidyverse’ (Wickham, 2016) and ‘tidytext’ (Silge and Robinson, 2016) packages, and for dimensionality reduction, we used the ‘umap’ package (Konopka, 2019).

## 3. Results

Table 1 shows the questionnaires included in the text analysis from our mapping review, the number of items in each questionnaire, the number of citations per article, and the labeled domain category.

**Table 1:** Published articles with trust questionnaires ordered by the number of citations in each category.

Paper	Items	Citations	Category
Jian et al. (2000)	12	1,139	Automation
Muir and Moray (1996)	9	1,014	Automation
Dzindolet et al. (2003)	1	967	Automation
Mcknight et al. (2011)	23	564	Automation
Madsen and Gregor (2000)	25	338	Automation
Singh et al. (1993)	12	278	Automation
Yagoda and Gillan (2012)	36	155	Automation
Merritt (2011)	6	145	Automation
Schaefer (2013)	40	140	Automation
Charalambous et al. (2016)	10	86	Automation
Chancey et al. (2017)	15	61	Automation
Körber et al. (2018)	1	58	Automation
Körber (2018)	19	54	Automation
Montague (2010)	28	52	Automation
Merritt et al. (2015)	10	51	Automation
Wiczorek and Manzey (2014)	1	50	Automation
Chien et al. (2014)	21	39	Automation
Goillau et al. (2003)	8	21	Automation
Albert et al. (2009)	1	20	Automation
Walliser et al. (2016)	1	18	Automation
Khalid et al. (2016)	14	15	Automation
Malle and Ullman (2021)	20	12	Automation
Moeckli et al. (2015)	12	11	Automation
Holthausen et al. (2020)	6	8	Automation
Salcedo et al. (2011)	20	5	Automation
Schneider et al. (2017)	6	5	Automation
Byrne and Marín (2018)	5	1	Automation
Gefen et al. (2003)	7	9,222	E-Commerce
McKnight et al. (2002)	3	5,981	E-Commerce
Bhattacherjee (2002)	7	1,835	E-Commerce
Delgado-Ballester et al. (2003)	8	1,202	E-Commerce
Sohaib and Kang (2015)	32	35	E-Commerce
Rotter (1967)	25	5,666	Human-Human
Rempel et al. (1985)	26	4,947	Human-Human
Mayer and Davis (1999)	38	2,863	Human-Human
Yamagishi and Yamagishi (1994)	24	2,782	Human-Human
Gefen and Straub (2004)	6	2,296	Human-Human
Larzelere and Huston (1980)	8	1,989	Human-Human
Yamagishi (1986)	5	1,750	Human-Human
Johnson-George and Swap (1982)	25	1,630	Human-Human
Wrightsman (1964)	13	526	Human-Human
Miller and Mitamura (2003)	2	380	Human-Human
Evans and Revelle (2008)	21	332	Human-Human
Frazier et al. (2013)	4	86	Human-Human
Goto (1996)	3	74	Human-Human
Allen et al. (2004)	7	33	Human-Human

Number of citations as of October 2021.

### 3.1. Word-level analysis

**Figure 1:**
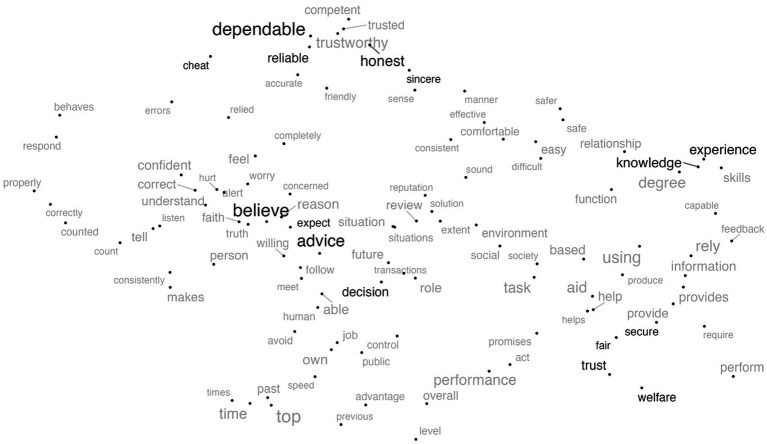
The UMAP two-dimensional representation of the questionnaire words. The words discussed in the manuscript as examples of specific themes are highlighted in black.

Figure 1 shows the relevant trust words gleaned from the questionnaires’ 626 items. The size of the word reflects its frequency; the bigger the word, the more often it occurs. The words are arrayed based on the UMAP dimensionality reduction of the word embeddings. The dimension of the GloVe word embeddings was 100 and using UMAP it was reduced to two. The choice of two dimensions was for visualization purposes. This approach has been used in several application and showed high performing results (Wang et al., 2021). The words near each other in this space are expected to have similar or complementary meanings. Some of the words create themes that directly map to different trust dimensions.

For example, in the middle of the figure, there is a cluster that includes “believe,” “expect,” “advice,” and “decision.” This area generally seems to be about truth reasoning and anticipating behavior and predictability. This contrasts with the bottom right cluster that focuses on fairness, and security and includes words like “fair,” “welfare,” “trust,” and “secure.” This is much more about how the behavior is valued. Also, the words in the upper right cluster include “experience,” “skills,” and “knowledge” which seem to characterize competence and ability. Finally, the upper left cluster includes words such as “dependable,” “reliable,” “competent,” “honest” which seem to characterize performance, and measure the integrity and reliability dimensions of trust. The upper left cluster also includes words like “cheat,” “honest,” and “sincere” which seem to characterize integrity. Whether or not these provide a comprehensive account of trust is the topic for other papers (Lee and See, 2004; Chiou and Lee, 2021; Malle and Ullman, 2021), but it certainly gives us an idea about the current and most common state of how researchers are measuring trust perceptions. Commonly, these dimensions of trust characterize trustworthiness in different objects, and this might explain why they are showing in certain clusters. For example, although not uniquely, integrity typically characterizes trust in humans whereas reliability characterizes trust in automation (Malle and Ullman, 2021).

### 3.2. Item-level analysis

**Figure 2:**
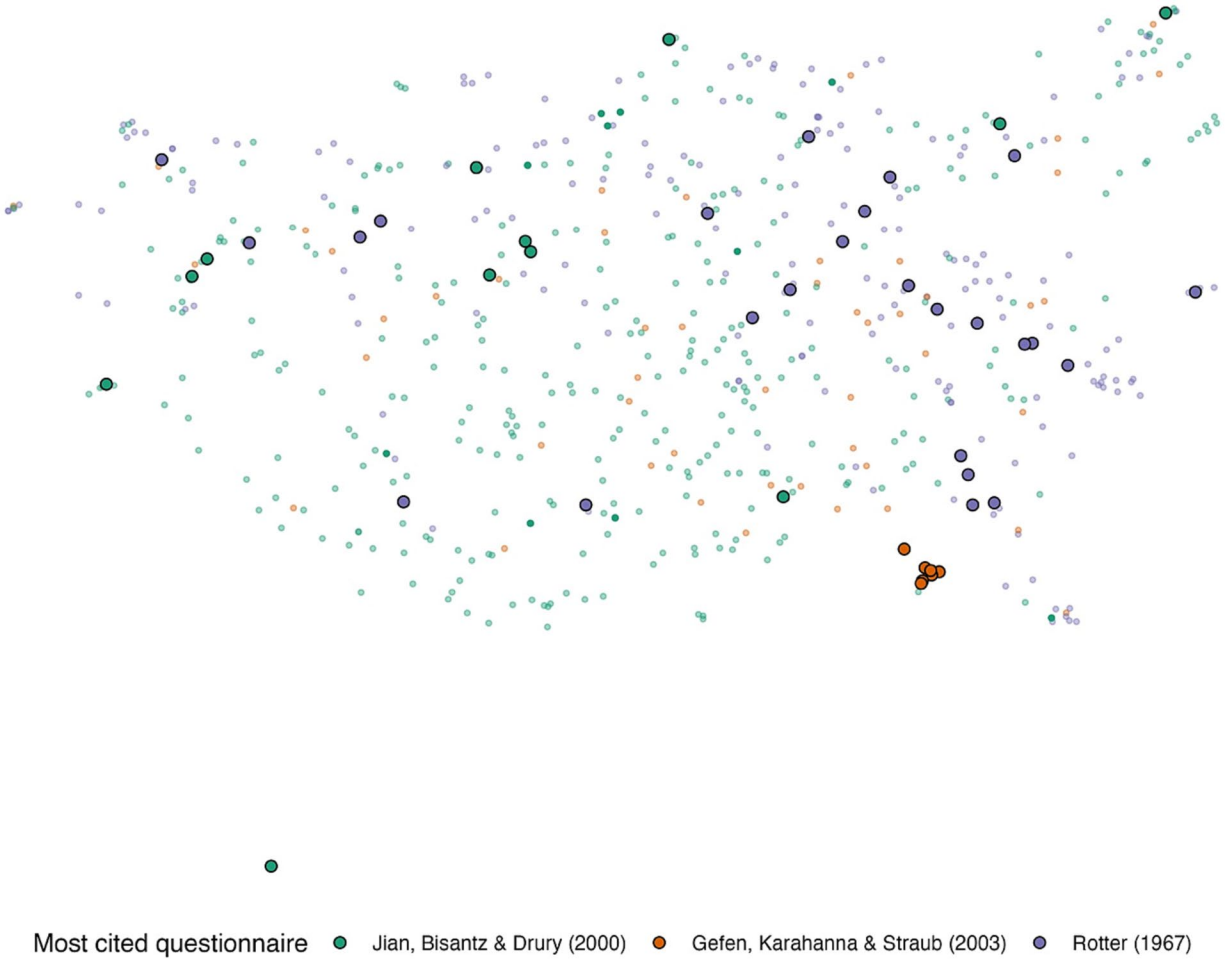
The UMAP two-dimensional representation of the embeddings of the questionnaire items. The most cited questionnaire in each category (Gefen et al., 2003 for e-commerce; Muir and Moray, 1996 for automation; Rotter, 1967 for human-human). Green, orange, and purple represent items in automation, e-commerce, and human-human trust questionnaires, respectively.

Figure 2 shows the UMAP representation of the items. The items from the most cited questionnaire in each domain are encircled and color-coded. Note that the figure shows *all* items of *all* questionnaires. The (Rotter, 1967) questionnaire was developed to measure human-human trust, the (Gefen et al., 2003) questionnaire was developed to measure trust in online shopping, and the (Jian et al., 2000) questionnaire was designed to measure trust in automation. Their different purposes are reflected in their placement in the semantic place.

Although very frequently cited, the questionnaire by Gefen et al. (2003) spans a small area, mainly because the items were similar and were developed to assess trust in an online vendor (i.e., “e-Commerce”) based on past experiences. The questionnaire items were framed as, “Based on my experience with the online vendor in the past, I know it is…,” with items assessing factors such as predictability and trustworthiness. On the other hand, the (Rotter, 1967) questionnaire has items that are widely spread across the semantic space. This is because it assesses dispositional trust through a large set of questions related to views of the future, attitudes toward society, and hypothetical ethical scenarios. Jian et al. (2000) trust in automation questionnaire has some items close in the UMAP space to those of Rotter’s interpersonal trust questionnaire; this is because it includes items that assess the reliability and dependability of automation and people.

Figure 2 showed that questionnaires vary in how their items spread across the semantic space. The questionnaire’s spread is an indicator of the breadth of the questionnaire, and the different dimensions of trust it covers. Table 2 shows the spread value of each questionnaire. Spread was calculated as the average Euclidian distance between the questionnaire’s items and the questionnaire centroid in the semantic space. As such, single-item questionnaires have a spread of zero.

**Table 2:** Questionnaires’ spread measured as the mean Euclidian distance between questionnaires items in the semantic space.

Paper	Category	Spread
Albert et al. (2009)	Automation	0
Dzindolet et al. (2003)	Automation	0
Körber et al. (2018)	Automation	0
Walliser et al. (2016)	Automation	0
Wiczorek and Manzey (2014)	Automation	0
Chancey et al. (2017)	Automation	3.2787649
Goillau et al. (2003)	Automation	9.8178918
Merritt (2011)	Automation	11.0535503
Singh et al. (1993)	Automation	13.3077262
Holthausen et al. (2020)	Automation	19.8532091
Merritt et al. (2015)	Automation	24.075077
Moeckli et al. (2015)	Automation	26.7285164
Yagoda and Gillan (2012)	Automation	26.8700501
Khalid et al. (2016)	Automation	26.9118744
Charalambous et al. (2016)	Automation	27.3718082
Byrne and Marín (2018)	Automation	32.1872312
Salcedo et al. (2011)	Automation	35.4811105
Muir and Moray (1996)	Automation	40.9128248
Schneider et al. (2017)	Automation	44.2913665
Madsen and Gregor (2000)	Automation	45.7479433
Jian et al. (2000)	Automation	52.9878988
Chien et al. (2014)	Automation	58.3499561
Malle and Ullman (2021)	Automation	63.6366837
Mcknight et al. (2011)	Automation	64.6944447
Körber (2018)	Automation	72.247291
Montague (2010)	Automation	117.608309
Schaefer (2013)	Automation	141.482416
McKnight et al. (2002)	E-Commerce	3.7473223
Gefen et al. (2003)	E-Commerce	4.047058
Bhattacherjee (2002)	E-Commerce	14.6113045
Delgado-Ballester et al. (2003)	E-Commerce	29.7256945
Sohaib and Kang (2015)	E-Commerce	73.1215945
Goto (1996)	Human-Human	0.1458184
Miller and Mitamura (2003)	Human-Human	1.1019821
Frazier et al. (2013)	Human-Human	7.5817419
Yamagishi (1986)	Human-Human	19.049358
Gefen and Straub (2004)	Human-Human	21.7186439
Wrightsman (1964)	Human-Human	23.8513333
Evans and Revelle (2008)	Human-Human	30.5084731
Larzelere and Huston (1980)	Human-Human	31.4059937
Rempel et al. (1985)	Human-Human	43.247883
Johnson-George and Swap (1982)	Human-Human	43.3757231
Allen et al. (2004)	Human-Human	44.0729489
Rotter (1967)	Human-Human	51.903995
Yamagishi and Yamagishi (1994)	Human-Human	85.8964228
Mayer and Davis (1999)	Human-Human	122.881738

### 3.3. Questionnaire-level analysis

**Figure 3:**
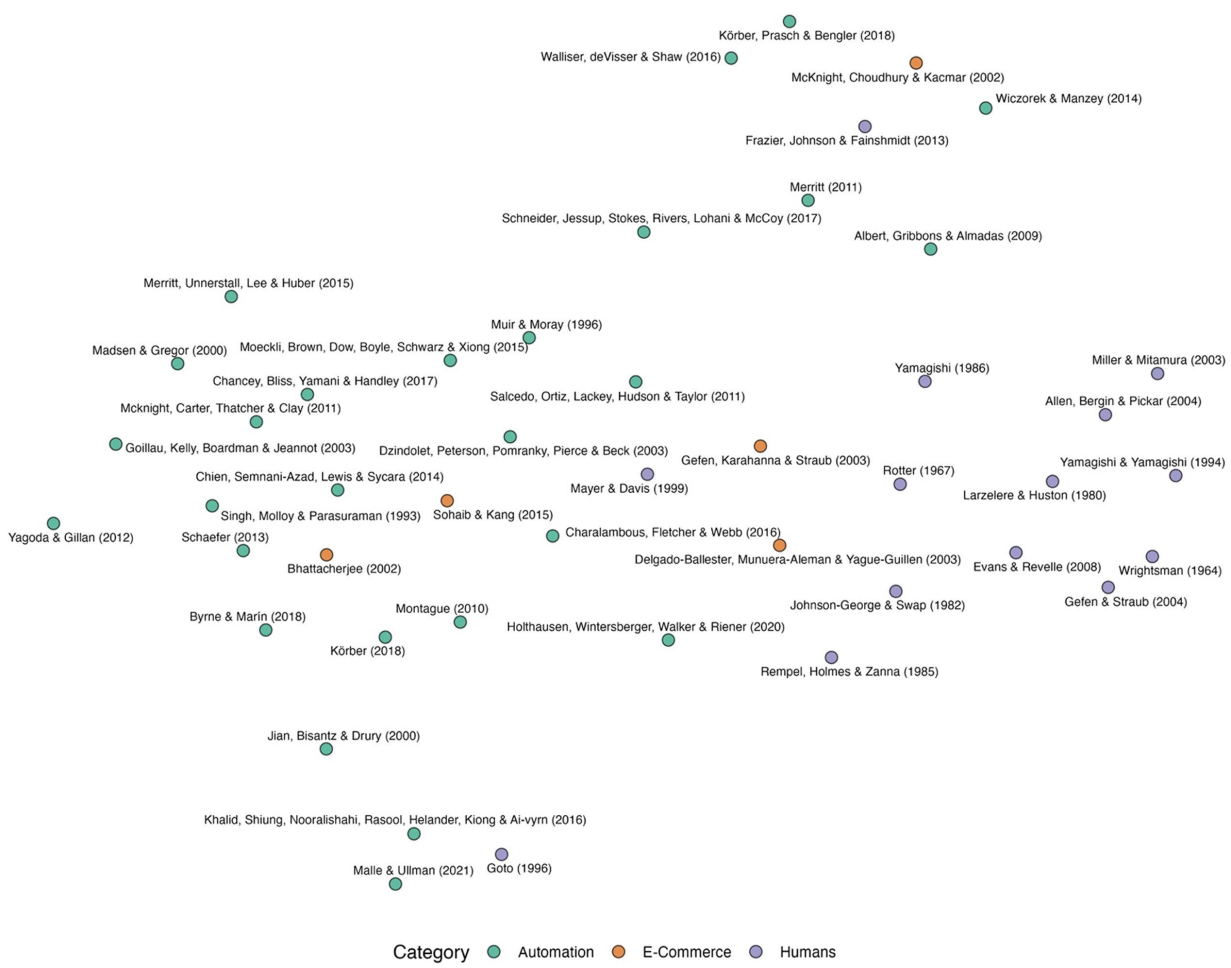
The UMAP two-dimensional representation of the questionnaire embeddings with domain category color-coded. Green, orange, and purple represent the automation, e-commerce, and human-human categories, respectively.

Figure 3 shows the UMAP semantic space representation of the questionnaires, which shows how the questionnaires relate to each other. The upper region of the semantic space is dominated by trust in automation questionnaires, whereas the bottom left region mostly consists of human-human trust questionnaires. The e-commerce questionnaires are spread across the entire space.

Questionnaires close to each other share terms or similar terms that make them close in the UMAP space. The bottom left cluster consisted of questionnaires about trust in humans and had many questions related to peoples’ behaviors such as “honesty” and “cheating” and how the person viewed “relationships” with and “personalities” of others. The questionnaires in the upper cluster mostly assessed efficiency, dependability, reliability, and safety in the specific domain that the questionnaire was developed. Finally, the questionnaires in the bottom right cluster commonly asked about the general tendency to trust (e.g., “I usually trust machines until there is a reason not to.)” The similarities and differences between these questionnaires can be further explored ls in the web app.

**Figure 4:**
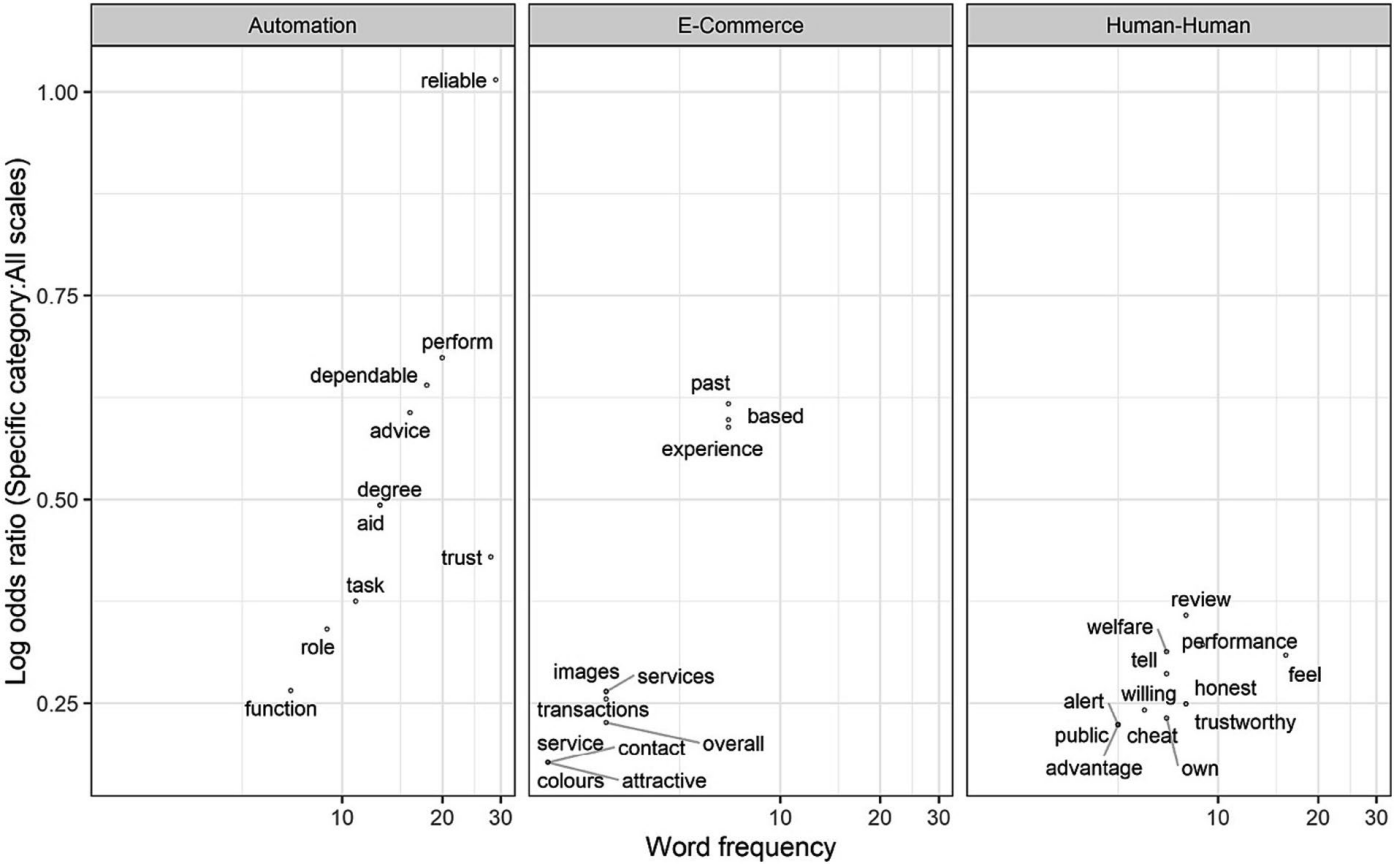
The log odds ratio of the 10 most common words in a specific category given all words in the questionnaires. The x-axis represents words frequency and is displayed on a logarithmic scale (base 10) to allow for the visualization of a wide range of values where the spacing between points increases exponentially as you move further right. The y-axis represents the log odds ratio of each word: the higher the word on the y-axis. The log odds ratio emphasizes words that are common in a specific category and relatively uncommon in others.

By calculating the log odds ratio of words in a specific category given all words used in all questionnaires, we identified the words that are most unique and distinguishing of trust across domains. Figure 4 shows the 10 highest frequencies of words in each domain (along the horizontal axis) and how unique these words are to each of the specific domains (along the vertical axis). Some words had tied frequencies, and thus the figure shows more than 10 words in e-commerce and automation domains. The figure shows that some words like “reliable” and “perform” were *more* unique to Automation, whereases words like “experience, or “transactions” were *more* unique to e-commerce, and words like “honest” or “cheat” were more unique to human-human. The log odds ratio emphasizes words that are *common in a specific category* and relatively *uncommon in others*, making them more frequent in their respective domains, though not exclusive to them.

This is different than the Term Frequency-Inverse Document Frequency (TF-IDF) metric. TF-IDF is used to assess the rarity or importance of a term in the document collection, while log odds ratio measures the association or relevance of terms to specific categories or classes. IDF focuses on the overall collection of documents, whereas log odds ratio emphasizes term relevance within specific categories or classes. For more details on the log odds ratio calculations see Appendix A.

### 3.4. Questionnaire composition

To help guide questionnaire selection, we assessed the questionnaire composition of domain-related words and layers of trust. The domain categories refer to trust in automation, e-commerce, or humans; the trust layers refer to dispositional, situational, or learned trust.

**Figure 5:**
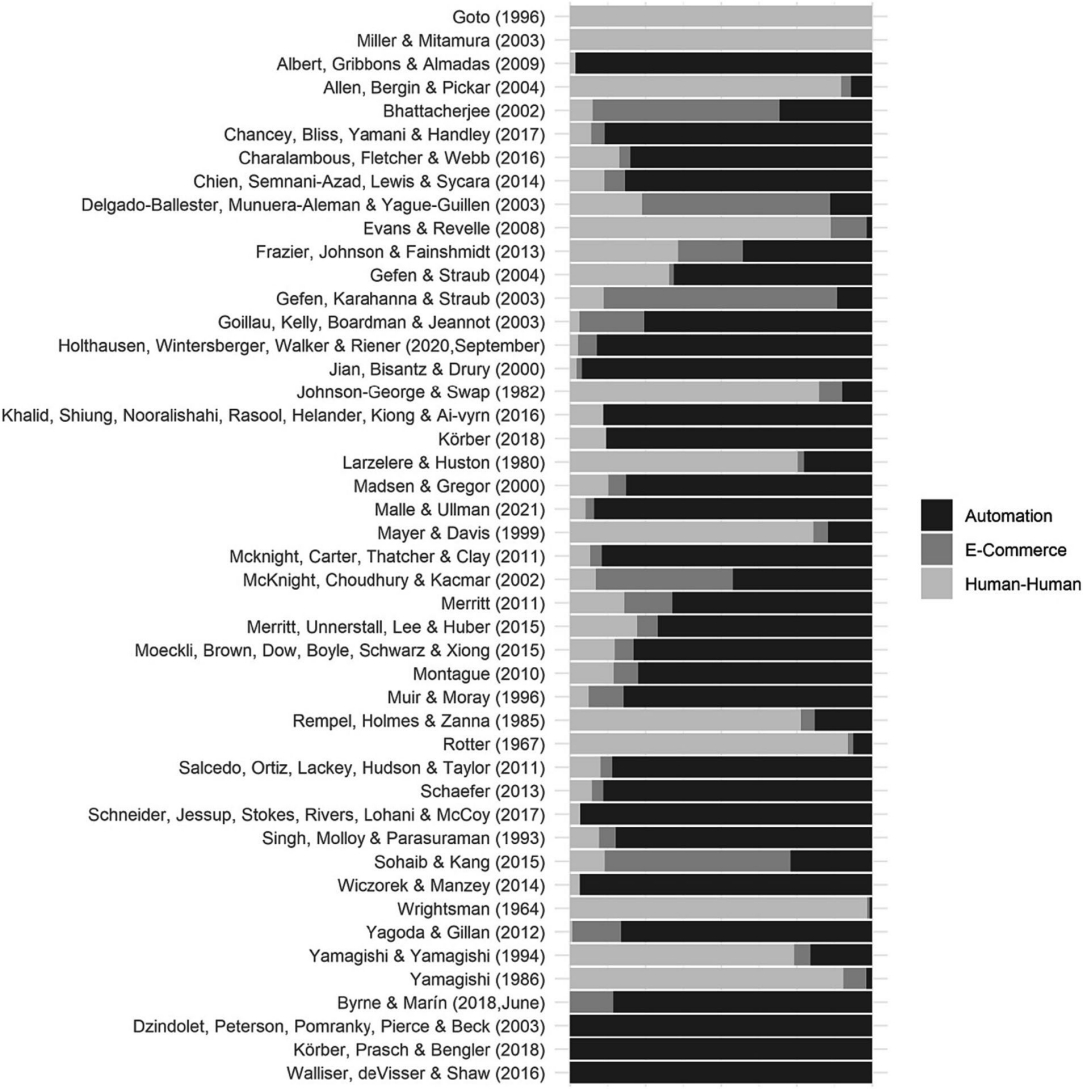
Trust questionnaires’ domain composition (containing words most related to which domains of trust).

To calculate the proportion of domain-related words in each questionnaire (e.g., the questionnaire can have 20% automation-related terms, 30% e-commerce-related terms, and 50% human-human-related terms), we used the log odds ratio results shown in Figure 4. The log odds ration compares the frequency of words in a certain domain to all words form all questionnaires. The questionnaires’ domain composition results are illustrated in Figure 5, where the questionnaires are ordered by the proportion of automation, e-commerce, and human-human content. For instance, Figure 5 shows that Goto’s (Goto, 1996) questionnaire is composed of 100% human-related words while the Delgado-Ballester et al. (2003) words involve a combination of each human, e-commerce, and automation-related words. In addition, we looked at words’ usage over the years and noticed that the language and terminology used in questionnaires underwent changes, but these changes were a result of the evolving nature of the domains rather than mere temporal shifts.

**Figure 6:**
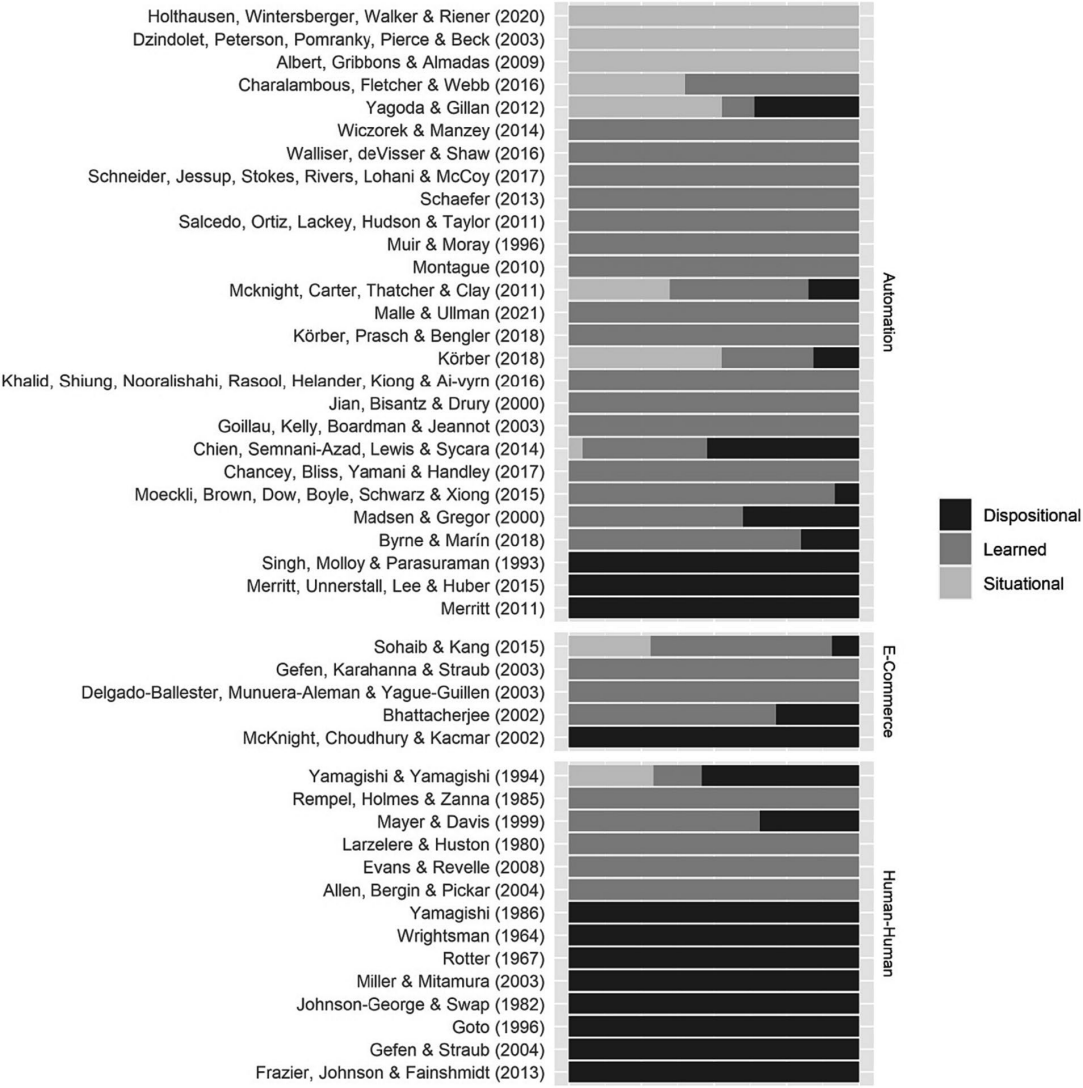
Trust questionnaires’ layers of trust composition of each questionnaire item.

Similarly, we calculated the proportion of each of the trust layers for each item. The items’ layers were coded by three researchers. One researcher led the labeling, while the other two provided reviewed the labels. The finalization of the labels occurred when all three researchers reached a consensus and agreed upon them. All items were categorized as either dispositional, learned or situational. The result is shown in Figure 6. Evans and Revelle (2008) is a questionnaire for measuring trust in humans and 100% of the questionnaire’s items are learned; all items assess trust based on previous experiences. The Rotter (1967) questionnaire also measures trust in humans, but its items are 100% dispositional, meaning all items assess a person’s innate tendency to trust. This is a starting point for selecting the questionnaire that best suits the research objective.

### 3.5. Trust lexicon

**Figure 7:**
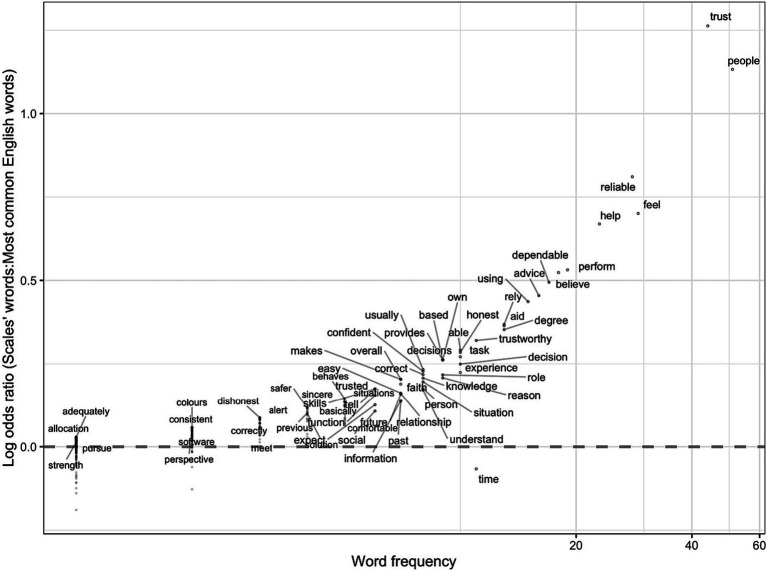
The log odds ratio of the words in the trust questionnaires to the words in the English language. The x-axis represents words frequency and is displayed on a logarithmic scale (base 10) to allow for the visualization of a wide range of values where the spacing between points increases exponentially as you move further right. The y-axis represents the log odds ratio of each word: the higher the word on the y-axis, the more unique the word is to trust questionnaires.

Figure 4 compared trust words in a specific domain category (i.e., automation, e-commerce, human-human) to trust words across all questionnaires. However, to identify trust-related words across domains, we compared them to the common words in the English language. We used a list of 5,000 frequent words from the Corpus of Contemporary American English (COCA) (Davies and Gardner, 2010). The corpus includes words from different genres; spoken, fiction, magazines, newspapers, and academic texts as shown in Figure 7. Words above the dashed line (*y* = 0), indicate words that are more common in the trust questionnaires than in common English usage, while words below the dashed line are more prevalent in common English than in the trust questionnaires. Not surprisingly “trust” and “people” occur frequently in the questionnaires and occur much more frequently in the questionnaires than they do in English usage.

**Figure 8:**
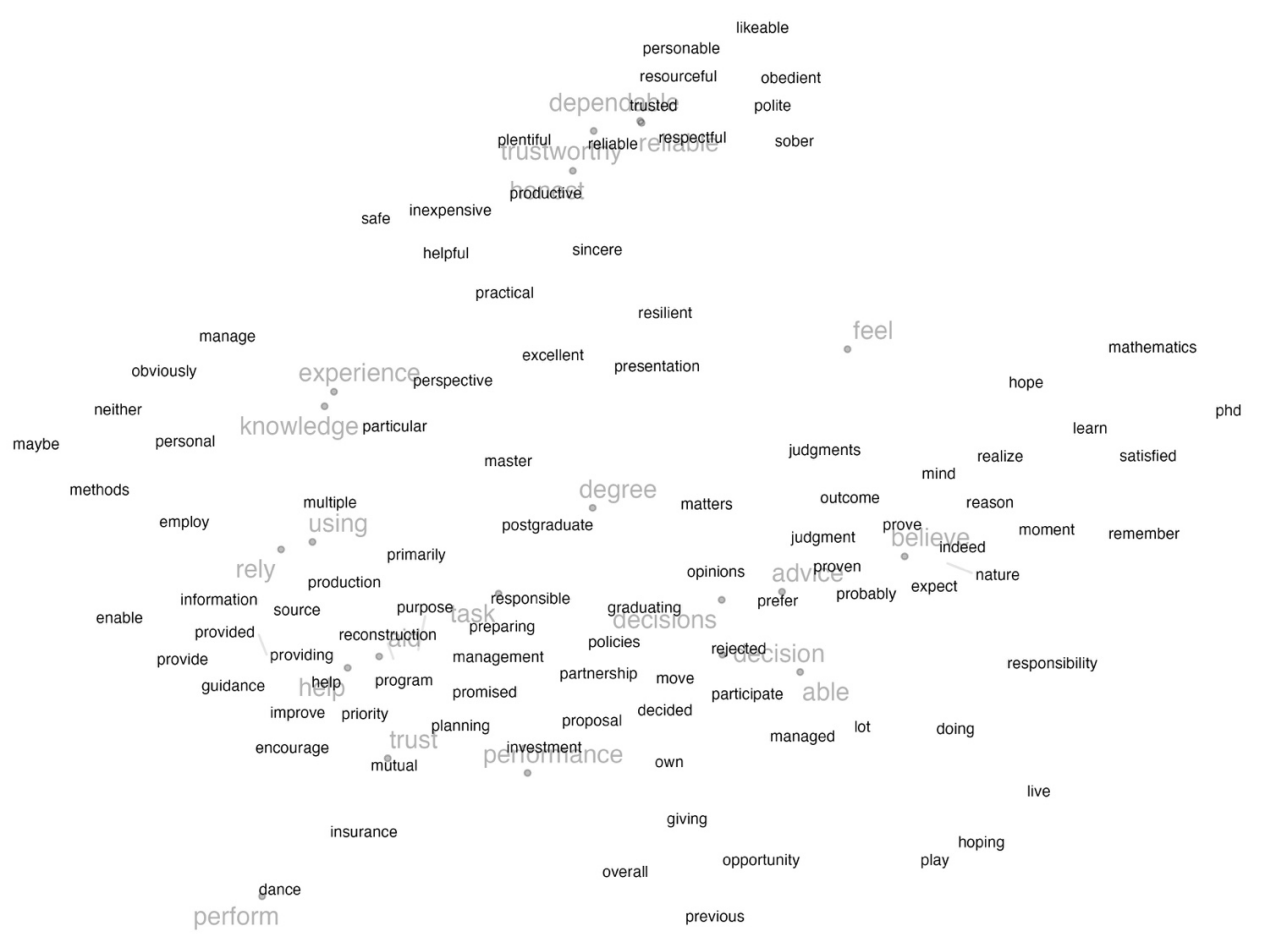
A lexicon of trust-related words. The words in light gray are the most common in the trust questionnaires, while the rest of the words are their closest neighbors in the high-dimensional GloVe embeddings.

Based on the 20 most unique trust words revealed in Figure 7, we created a trust lexicon shown in Figure 8. For each of the 20 words from Figure 7, we extracted the five closest neighboring words in the high-dimensional GloVe embeddings space using the cosine distance. Before extracting the five closest words, we conducted the same data cleaning steps described in the methods on the GloVe words, to avoid words like “the” and “from.” The 20 most unique trust words from the questionnaires are shown in light gray, while the other words in dark gray are their closest neighbors.

## 4. Discussion

The results of the word-embedding text analysis identified the most common words used to assess trust, revealed semantic similarities and differences across the trust questionnaires, and provided a detailed comparison of the questionnaires’ composition based on domain and trust layers. The results were implemented in an interactive web application that allows further exploration of the analysis. Particularly, the word-level and item-level results can support further questionnaire development and the questionnaire-level results can aid questionnaire selection given the research objective for measuring trust, and consideration of the trusting context.

### 4.1. Word-level analysis: common words and trust lexicon

The word-level analysis allowed us to identify the frequently used words to measure trust. Common words included “dependable,” “experience,” and “advice.” The neighboring words in the semantic space also had similar or complementary meanings such as “reliable,” “knowledge” and “helpful,” respectively. Word clusters shown in Figure 1 can be mapped to trust dimensions identified by Malle and Ullman (2021). “Dependable” and “reliable” are closely situated next to each other, which can be mapped to the performance dimension of trust, whereas “secure” and “fair” show the moral dimension of trust. The themes revealed by the most frequent words were consistent with literature on trust dimensions (i.e., integrity and competence) (Malle and Ullman, 2021). When categorized into trust in automation, humans, and e-commerce, more specific themes emerged (e.g., reliability and dependability to describe automation. Honesty and sincerity to describe humans, and prior experiences and services to describe e-commerce).

Understanding the words associated with trust questionnaires can be helpful in different ways. The identified trust-related words can be used as a single-word trust assessment tool, e.g., by asking subjects to rate how well these words describe the system of interest. The trust-related words can be descriptors used in card sorting tasks to measure trust, similar to the microsoft desirability toolkit (Benedek and Miner, 2002). In addition, these words can be used as a basis robust or more precise instruments for measuring trust. For example, the Jian et al. (2000) scale development process consisted of three key steps: collecting comprehensive list of trust-related words, assessing word similarity through a questionnaire, and rating the similarity of word pairs through a paired comparison study. The resulting words were then clustered and used to construct the trust scale. This process can be intensive and tedious. Leveraging the identified words can help us study their similarity without subjective bias or intensive manual effort, which could make future instrument development more robust and precise.

By examining the most common words in each domain, we found similarities and differences in what questionnaires typically use to describe trust in automation compared to trust in humans. This can be because some forms of automation are more human-like, such as anthropomorphic agents or virtual humans, hence questionnaires involving more human-like qualities in these research contexts may be more appropriate (Lankton et al., 2015). Furthermore, there are certain trust dimensions that are relevant to both the human and the automation such as integrity and competence, which can explain the similarities.

The words’ semantic space revealed by this analysis was used to create a trust lexicon. Word sentiment lexicons can estimate people’s emotional state (Tausczik and Pennebaker, 2010) and attitudes on social media (Pang and Lee, 2008) or in conversations (Li et al., 2020). Word sentiment lexicons are typically created through tedious manual labeling of each word in the dictionary, which produces a sentiment rating for each word. But when word embeddings are combined with dimensionality reduction techniques, they reveal correlations between words and how they might relate which can expedite the development of lexicons. The lexicon resulting from our analysis can build on and improve similar lexicons of trust-related words (Mohammad and Turney, 2013). However, our approach relied solely on the machine learning algorithms to identify similarities and difference between words used in trust assessment questionnaires. While machine-learning-led approaches are helpful and efficient, they have inherent limitations. For instance, in Figure 8 we can see the proximity of words like “degree,” which is commonly used in trust questionnaires, to words like “postgraduate” which is irrelevant to trust. Future work might explore a mixed-initiative approach to lexicon development leveraging machine learning efficiency and human expertise (Alsaid and Lee, 2022; Alsaid et al., 2023). This synergy can provide more robust and accurate results in various natural language processing tasks.

### 4.2. Item-level analysis: items’ spread and trust characteristics

The item-level analysis showed that some questionnaires had items close to each other while items in other questionnaires were more dispersed. The distribution of the items was linked to the variation of the trust characteristics being evaluated by different items in each questionnaire (e.g., reliability, performance, cheating, prior experience, etc.); the greater the spread of a questionnaire’s items, the more layers and characteristics are captured.

In general, the human-human questionnaires were broadly distributed. Because, in line with the questionnaire-level results, items in this domain tended to assess a wide range of human characteristics in different hypothetical scenarios. This implies that these questionnaires used words from varying contexts, which would explain their spread in the semantic space. On the other hand, some questionnaires’ items were contained in a very small area in the semantic space. One illustrative example is the most cited e-commerce questionnaire (Gefen et al., 2003); the questionnaire’s items assess similar characteristics (and thus used closely related words) of online vendors such as “reliability,” “honesty” and “trustworthiness.”

In summary, the distribution of the questionnaire items in the semantic space can reflect the variety of trust dimensions being measured – the more dimensions the trust questionnaire captures, the more spread the items are. Therefore, one important consideration when selecting a trust questionnaire is the spread of its items. If the research question requires evaluating a specific quality that is associated with trust (e.g., reliability), then researchers could pick questionnaires with items that are closer together in the semantic space, whereas if the research questions require evaluating multiple qualities associated with trust (e.g., prior experience, performance, deception), a more spread questionnaire is likely more appropriate. Careful examination of the questionnaire and its constituent items is necessary.

### 4.3. Questionnaire-level analysis: domain- and layer- based selection

At the questionnaire level, the results revealed three main clusters, one consisting of mainly human-human trust questionnaires, and two containing a mix of trust in automation and trust in e-commerce questionnaires as shown in Figure 3. Questionnaires assessing a person’s trust in other people were typically broad and contained diverse items assessing learned, dispositional, and situational trust through hypothetical scenarios, general views about the world, and the overall tendency to trust others. Questionnaires assessing trust in automation had two common themes with those assessing trust in e-commerce. Because after removing domain-specific words (e.g., “website” and “vendor” for e-commerce and “robot” and “automation” for automation), items of both domains were largely similar. One theme focused on assessments of reliability, accuracy, and trustworthiness of a system, while the other theme focused on the general tendency of people to trust or not trust new technologies. This explains the proximity in the semantic space, nonetheless, questionnaires developed for trust in automation might not be appropriate to assess trust in e-commerce and vice versa. Depending on the context and the research question, one theme or a combination of themes might be more appropriate, and researchers should carefully consider the aspects of trust being evaluated by each questionnaire (Kohn et al., 2021).

The questionnaire composition analysis provided a descriptive map for questionnaire selection based on layer and domain. In the layer composition analysis, questionnaires were summarized based on constituent items’ layers whereas in the domain composition analysis, questionnaires were summarized based on their constituent words uniqueness to certain domains. This provides an overview of each questionnaire’s composition and enables their comparison, however, aggregating and summarizing data in this way may result in overlooking certain details, like the overlaps highlighted in Figure 2. For instance, while the Rotter (1967) questionnaire exhibited large overlap in Figure 2 and high spread in Table 2, it appeared to be mostly composed of human-human words in Figure 5. This similar to reducing experimental data to mean values: while it might not capture every detail, it provides a useful overall summary.

### 4.4. Practical implications: questionnaire selection guidelines

The questionnaire composition analysis provided further clarity on how the myriad trust questionnaires compare to one another and can thus serve as initial guidance for selecting a questionnaire. Here, we outline general guidelines and considerations for the trust questionnaire selection process: identifying the domain and layer, and considering items’ dispersion, and evaluating the tradeoff between number of items and sampling frequency.

After carefully defining the research questions and the underlying hypotheses, the researcher needs to identify *the domain* in which trust is being measured. This is important because trust questionnaires are typically developed to measure trust in a specific context and the way trust is characterized varies from one domain to another (Lee and See, 2004; Lewis and Weigert, 2012). This was evident in the word-level analysis in Figure 4: the words used to describe trust differed across domains. For example, Lee and Moray (1992) conceptualization of purpose (e.g., role), process (e.g., dependable), and performance (e.g., reliable) dimensions of trust in automation was apparent in some of the words, as well as the concept that trust may also have more moral dimensions (Mayer et al., 1995; Sheridan, 2019). Another important consideration is the attributes of the trustee. In Figure 5, we provided a questionnaire composition map to show what percentage of each questionnaire included words most unique to trust in automation, human-human, or e-commerce. The figure showed that some e-commerce questionnaires had a percentage of automation-related words, and some automation questionnaires had a percentage of human-human related words. In instances where the trustee is more human-like, it might be appropriate to select a questionnaire with higher human-human percentage. Nonetheless, this was based on the objective quantitative analysis, researchers should carefully assess whether or not a questionnaire is appropriate for measuring trust in a certain domain.

Moreover, identifying the *layer* of trust is important; whether the researcher is trying to assess people’s general propensity to trust (i.e., dispositional), trust in a specific situation (i.e., situational), or trust based on previous experiences (i.e., learned). In Figure 6, we provided a map for understanding the composition of the questionnaires. Based on the research questions, the selected questionnaire items can be dispositional, learned, situational, or a combination (Merritt and Ilgen, 2008). It is important in this step to understand the nature of each trust layer. Measuring dispositional trust would be most appropriate for studies of individual differences, particularly when measuring trust across different cultures, as people from different cultures may have different perceptions of trust. Moreover, learned trust would be for studies of how interactions with an agent affect trust, and situational trust would be for measuring trust in a specific event. For example, if researchers are interested in evaluating the users’ trust in automation in specific of interactions, a questionnaire that mainly consists of situational trust items would be suitable [e.g., Holthausen et al. (2020)] whereas if they wish to assess the persons’ propensity to trust, a questionnaire like that mainly consists of dispositional trust item [e.g., Merritt (2011)] would be better suited.

Furthermore, the results revealed another element of the questionnaires’ semantic characteristics and selection criterion: questionnaire items spread. The *spread* of the questionnaire items is an important criterion of selection: whether the research question and nature of the study focus on one or a few of the dimensions of trust (e.g., purpose, process, or performance information for forming a person’s trust in automation (Lee and See, 2004), or rational and relational dimensions of trust in others (Lewis and Weigert, 2012)). This can be qualitatively determined by visually assessing the specific questionnaire items’ distribution across the semantic space, or quantitatively by the spread values in Table 2 that were calculated as the mean of Euclidian distances from a questionnaire’s centroid in the semantic space. When selecting a questionnaire, if researchers are interested in trust as a moderator or control variable (Fuchs and Diamantopoulos, 2009) or only focusing on a single aspect of trust (e.g., the performance of a particular automated system), then picking a narrower spread of trust scale can be appropriate (e.g., Chancey et al. (2017) for trust in automation which focuses on the ability and dependability dimensions of trust, Goto (1996) for trust in humans which measures trust relative to social distance, or McKnight et al. (2002) for trust in e-commerce which measures tendency in particular). If researchers are interested in assessing various characteristics and layers of trust in the study, then a broader spread of trust scale should be considered (e.g., Schaefer (2013) for trust in automation, Mayer and Davis (1999) for trust in humans, or Sohaib and Kang (2015) or e-commerce).

In addition, evaluating the trade-off between the *number of questionnaire items* and *sampling frequency* is critical (Kohn et al., 2021). If trust needs to be measured multiple times for its dynamic characteristic, using a few or single itemed questionnaire might provide a quick trust measurement and minimal interruptions to the continuity of a study participant’s experience (Körber, 2018). However, one item might be limited and not measure the different dimensions of trust (Lee and See, 2004). If the research objective requires a more detailed assessment of trust, then multi-item questionnaires are recommended [e.g., Yagoda and Gillan (2012)]. This is particularly important in situations where different layers of trust need to be measured at different times of a study (i.e., dispositional trust before the study, situational trust during the study). In combination with the questionnaire composition analysis, the researcher can make an informed decision regarding the questionnaire selection with the right number of questions that meets the research needs.

Finally, the supplemented web app implementation provides an interactive interface to compare, contrast and select the questionnaire most appropriate based on the considerations provided above. For a more detailed explanation and description of how to use the app for questionnaire exploration and selection see Appendix B.

### 4.5. Limitations and further research

This study has several limitations. First, some of the identified words as part of the trust lexicon (e.g., “feel” or “believe”) may be more an artifact of the measurement method and our ability to elicit self-report from lay-people (i.e., not trust scholars) through a scaled question (e.g., “how much do you feel…”). Framing questions as such is a common means to measure and quantify attitudes (Michael, 1979). Because attitudes have emotional, cognitive, and behavioral dimensions, asking questions about feelings enhances construct validity (Schwarz and Bohner, 2007) Nonetheless, trust is fundamentally an attitude, and not an intention, feeling, belief, or behavior (Lee and See, 2004).

Second, in the questionnaire composition analysis, we show what each questionnaire measures, in terms of domain and layers of trust, while remaining agnostic as to whether or not these questionnaires measure them well. Researchers should self-assess and investigate further the validity of each measure for their research task at hand, as is the standard practice of scientific rigor. Furthermore, the questionnaire composition analysis does not precisely reflect the effect of the number of items. That is, a single-item questionnaire would be 100% dispositional, learned, or situational. But that does not necessarily mean that it is the best questionnaire to measure that specific trust layer. In addition, the trust layer categorization was based on the specific questionnaire’s purpose, however, the same question can be used to assess different layers of trust depending on the context and the time it was administered (i.e., before, during, or after an interaction) (Merritt and Ilgen, 2008).

Third, our categorization of the trust questionnaires’ domains is rather generic. For example, some automation trust questionnaires are targeted at trust in automated vehicles specifically whereas others are targeted at trust in automation in general. We tackle this in the analysis by removing system-specific terms (e.g., robot, vehicle, website), however, questionnaire specificity remains an important consideration in questionnaire selection. A questionnaire that assesses trust in an assistive robot might have more human-like questions that do not necessarily translate to trust in an automated vehicle.

Fourth, the similarity found between the trust in automation and trust in e-commerce questionnaires may have been due to having similar theoretical origins; both categories assess trust in some type of technology or trust in an entity mediated by technology (Ghazizadeh et al., 2012) – to understand trust-related decisions such as reliance or purchasing. We are not claiming that this is a novel finding, and indeed assessing the history of these questionnaires would lead to a similar insight. However, our approach reveals this relationship through a quantitative, systematic analysis that shows researchers across multiple domains have similarly operationalized the construct of trust.

Fifth, our approach to characterizing the similarities and differences between questionnaires was data-driven. Data-driven approaches have been proven useful in expanding knowledge and extracting scientific relationships years in advance of their discovery (Tshitoyan et al., 2019). Yet, trust is a complex, multifaceted construct and future work should incorporate a theory-driven approach to safeguard the theoretical underpinnings of trust, expand trust theory, and build on existing measures (McCroskey and Young, 1979; Long et al., 2020).

Sixth, our analyses leveraged one method of text analysis: word embeddings. Although this approach has demonstrated effectiveness and has been widely employed in various applications, it is important for future studies to explore alternative methodologies, such as topic modeling. Hierarchical topic modeling, in particular, can prove valuable, as it preserves the structure of the word-item-questionnaire and offer a different perspective on the data.

Seventh, we are potentially missing some questionnaires – a more comprehensive review might have revealed more relevant questionnaires, such as studies that focus on information credibility that may be related to trust (Renn and Levine, 1991; Fogg and Tseng, 1999; McCroskey and Teven, 1999). However, the methods used in this paper are scalable and could be easily applied to an expanded corpus if new questionnaires are developed or to explore a broader conception of trust. Futhermore, one limitation of the study is the inclusion of questionnaires in the corpus that were developed *ad hoc*, relying primarily on face validity, or were not empirically validated (Gutzwiller et al., 2019). This may lead to potential issues for item and questionnaire selection.

Finally, one important limitation of this study is that it only included questionnaires developed in English. Excluding trust questionnaires that might have been developed in another language has important implications for advancing trust theory and methods of trust measurement across languages and cultures, but also for generalizability of the trust lexicon.

## 5. Conclusion

This study demonstrates the potential of text analysis in understanding trust questionnaires in different contexts, which provides a systematic method to quantify similarities and differences for further survey development and questionnaire selection.

The analyses conducted were at the word, item, and questionnaire levels. Each highlighted important considerations of questionnaire development and selection. The word-level analysis showed the most common words and themes that emerged from the trust questionnaires literature and produces a trust lexicon. This has implications for questionnaire development and understanding of trust in conversational speech and public attitudes on social media (Pang and Lee, 2008; Li et al., 2020). Furthermore, the item and questionnaire analyses provided higher-level insights into questionnaire items composition, and questionnaire items spread across the semantic space, both of which are important considerations for questionnaire selection.

While this study focused on text-based trust questionnaires, this approach can be extended to more specific domains; such as estimating drivers’ trust in self-driving vehicles through speech using the developed trust lexicon, similar to previous work on emotion classification (Aman and Szpakowicz, 2007) and the analysis of open-ended survey responses (Lee and Kolodge, 2018).

Overall, word-embedding text analysis is a useful way to understand the sentiments and emotions associated with words. The resulting semantic space of trust words provides a way to compare and select trust questionnaires. In addition, the resulting lexicon of trust-related words can be used in natural language processing to understand trust attitudes through conversations between people, and between people and technologies in different domains.

## Data availability statement

The original contributions presented in the study are included in the article/Supplementary material, further inquiries can be directed to the corresponding author.

## Author contributions

AA and JL conceived of the presented idea. AA and ML conducted the questionnaires literature review. ML and EC contributed significantly to the planning and direction of the paper. AA conducted the analysis and developed the web app. All authors discussed the results and contributed to the final manuscript.
