# Supplementary material for: Measuring trust: a text analysis approach to compare, contrast, and select trust questionnaires
Source: Front Psychol. 2023 Nov 15;14:1192020. doi: 10.3389/fpsyg.2023.1192020 (PMC10684734; doi:10.3389/fpsyg.2023.1192020)
Supplement: Supplementary file 1 [file Data_Sheet_1.PDF]

Appendix A:
Mathematical Formulas

The log odds ratio

For the log odds ratio calculations, we used the “bind_log_odds” from the “tidytext” package in R (Silge & Robinson, 2016, 2019). The calculations were derived from (Monroe, Colaresi, & Quinn, 2008).
The log odds of word w in document i is defined as:

$$\log O_w^i = \log \frac{f_w^i}{1 - f_w^i}$$

And the log odds ratio of for comparing the usage of word w across document i and j is:

$$\log \frac{O_w^i}{O_w^j} = \log \frac{f_w^i}{1 - f_w^i} / \frac{f_w^j}{1 - f_w^j} = \log \frac{f_w^i}{1 - f_w^j} - \log \frac{f_w^j}{1 - f_w^j}$$

When a word is only present in one of the documents, the odds ratio dominator will be zero. A solution is to add a small ε term to the dominator.

$$\tilde{f}_w^i = f_w^i + \varepsilon$$

Uniform Manifold Approximation and Projection (UMAP)

In this paper we used the “umap” function from the “umap” package in R (Allaoui, Kherfi, & Cheriet, 2020; McInnes, Healy, Saul, & Großberger, 2018). The number of neighbors (n_neigbors) hyper parameter varied. For the word-level analysis, we used the package’s default, for the item analysis we used n_neighbors of 40, and for the questionnaire we used n_neighbors of 17. The n_neighbirs values were selected subjectively based on the performance of the umap clustering at each n_neighbor value. No other hyperparameters were changed.

UMAP uses an exponential probability distribution to measure the similarity between two data points in a high dimensional space:

$$p_{ij} = \exp\left(-\frac{d(x_i, x_j) - p_i}{\sigma_i}\right)$$

Where d is the distance between points x_i and x_j, and p_i is the distance between x_i and its nearest neighbor.

In order to construct the low dimensional representation, UMAP uses a probability measure for the distance between points in low dimension:

$$q_{ij} = (1 + a(y_i - y_j)^{2b})^{-1}$$

And a binary cross entropy function as a cost function:

$$CE(P, Q) = \sum_i \sum_j [p_{ij} \log(\frac{p_{ij}}{q_{ij}}) + (1 - p_{ij}) \log(\frac{1 - p_{ij}}{1 - q_{ij}})]$$
