# Supplementary material for: Measuring trust: a text analysis approach to compare, contrast, and select trust questionnaires
Source: Front Psychol. 2023 Nov 15;14:1192020. doi: 10.3389/fpsyg.2023.1192020 (PMC10684734; doi:10.3389/fpsyg.2023.1192020)
Supplement: Supplementary file 2 [file Data_Sheet_2.PDF]

Appendix B:
Trust questionnaires explorer

The trust questionnaire explorer is a web app implementation of the word embedding text analysis of trust questionnaires. It provides an interactive interface for researchers to closely explore and examine the semantic space of trust questionnaires, their constituent items, and words.

This appendix provides a step-by-step clarification of how to use the app with the questionnaire selection guidelines in mind. The trust questionnaire explorer can be accessed at: https://areen.shinyapps.io/Trust_explorer/

Overview of the trust questionnaire explorer:

The trust questionnaire explorer consists of three main tabs: the questionnaire, item, and word level. A tab can be selected by clicking on the top of the explorer shown in Figure 1.

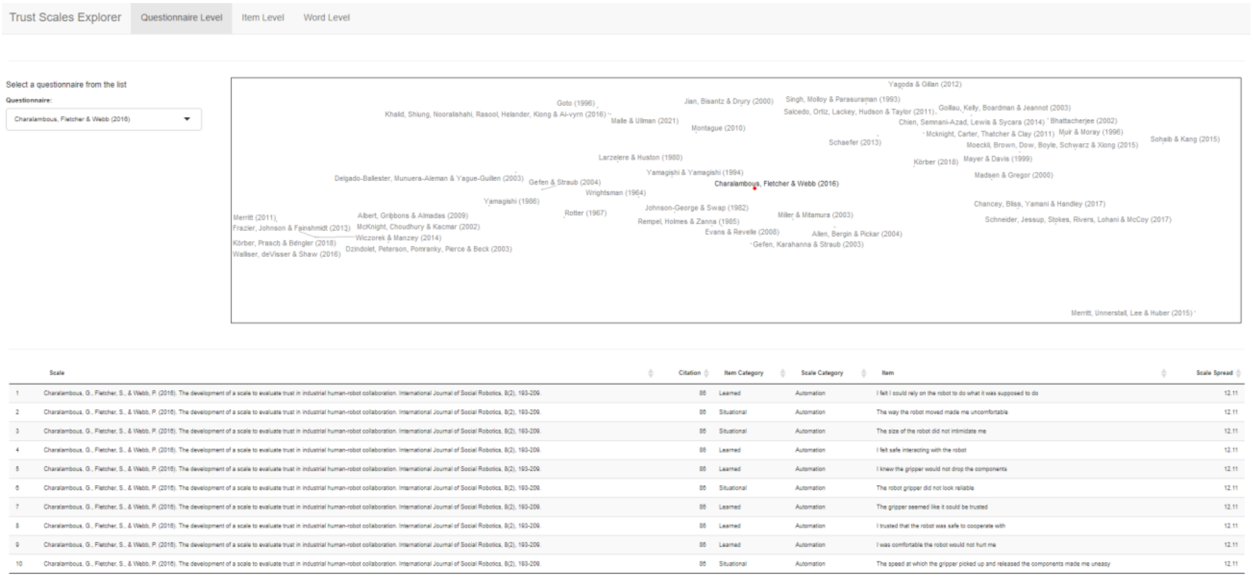

Figure 1. An overview of the trust explorer. The three tabs are highlighted at the top: the questionnaire, item, and word levels.

Questionnaire level:

In the questionnaire level tab, the user can select a specific questionnaire and explore its items, citations, the spread and the items' trust layer. Figure 2 shows an example. This tab mainly helps researchers find all questionnaire and their items in one place where they can use it in research and studies.

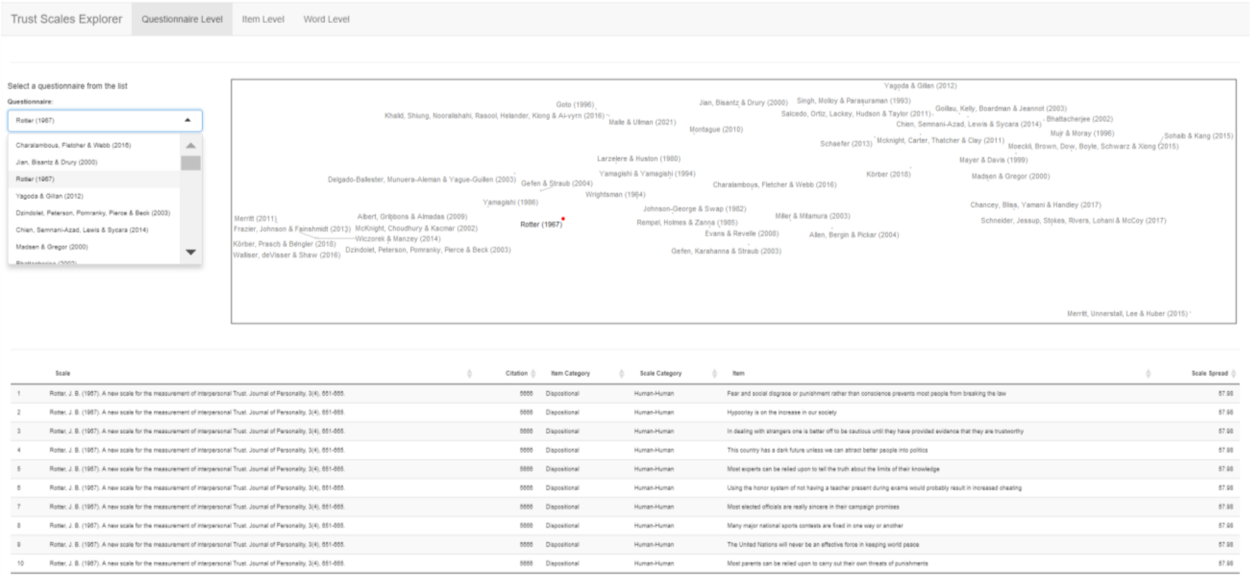

Figure 2. The questionnaire level tab. A questionnaire can be selected from the drop-down menu. The questionnaire location in the umap space will then be highlighted, and the questionnaire items, category, citations, and spread will be shown in the table below.

Item level:

The item level tab provides a more detailed view. In the middle, it shows the umap space of all items in all questionnaires. When first opened, it is set to show the Charalambous, Fletcher & Webb (2016) questionnaire. By brushing points in the umap space, items of other questionnaires can be explored. Figure 3 shows an example. The brushed items are shown in the table. They are color-coded by questionnaire. Furthermore, the rest of each questionnaire's items are highlighted and color-coded similarly to the brushed points.

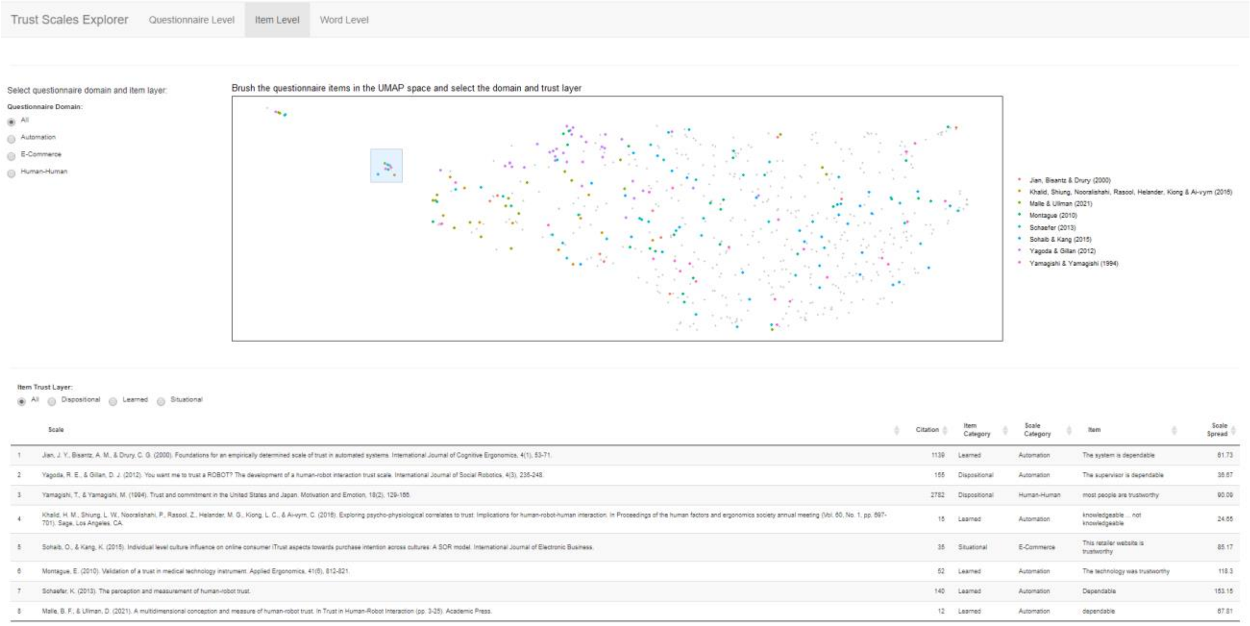

Figure 3. The item-level tab shows the items umap space in the middle. By brushing the items umap space in the middle, the items of the questionnaires will be highlighted and displayed in the table below.

Furthermore, the questionnaire domain can be selected from the radio buttons on the left of the umap figure. This will narrow down the questionnaires shown. For further filtering, the item trust be selected from the radio buttons above the table. Figure 4 shows the results after selecting 'automation' questionnaires only, and 'learned' trust layer.

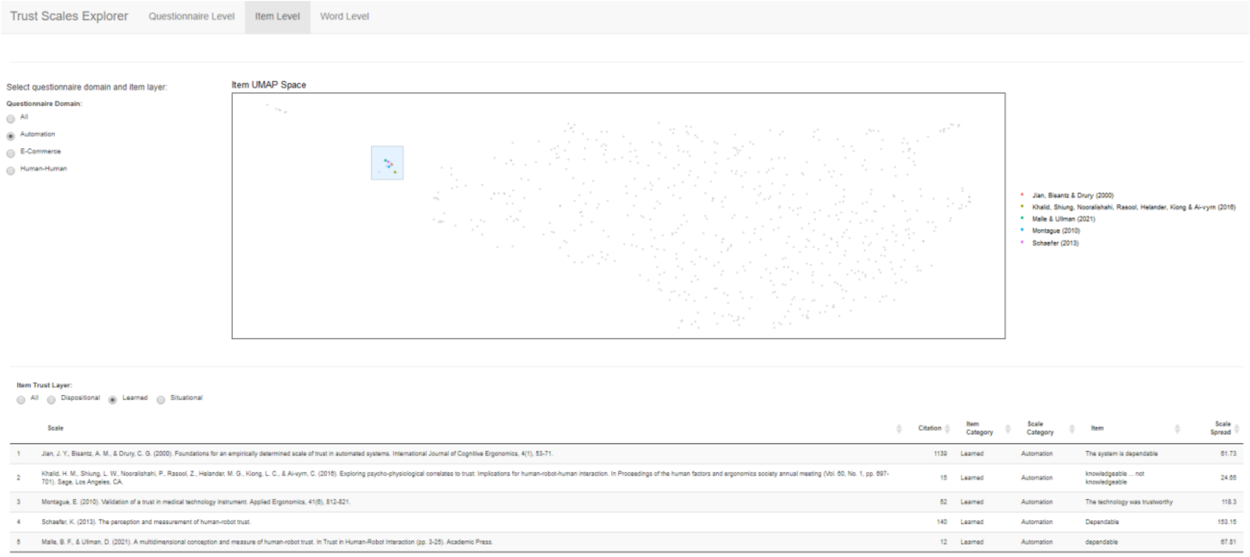

Figure 4. Item umap space filtering based on questionnaire domain and item trust layer.

Word level:

The word level tab is similar to the item level tab. Words in the umap space can be brushed, the table will show the items in which the words appeared as shown in Figure 5. This provides context to how the words were used to assess trust.

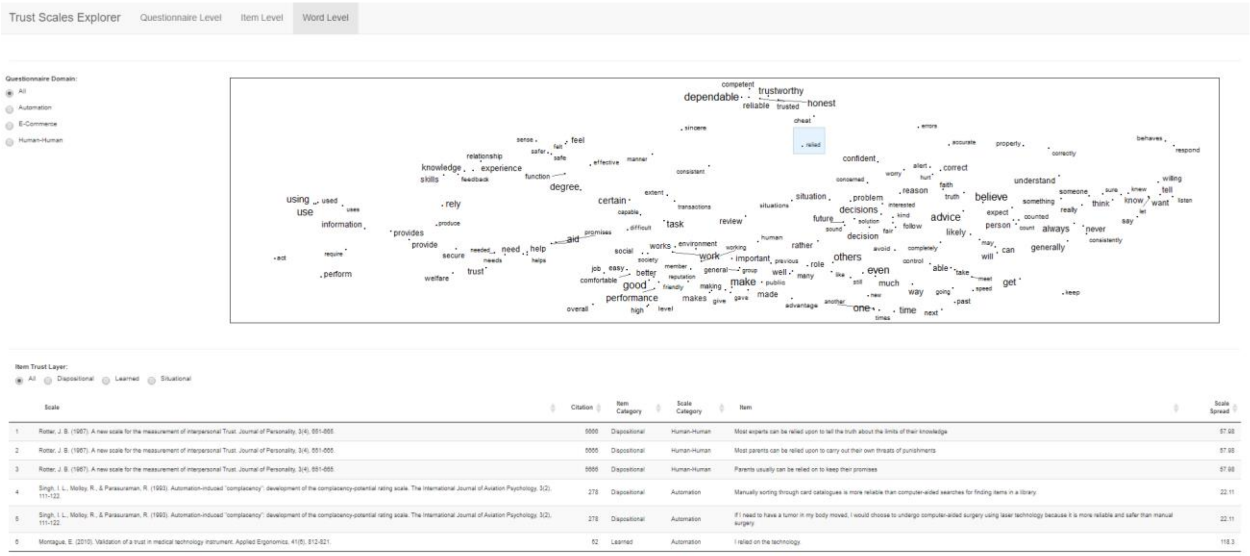

Figure 5. The word-level tab shows the umap space of the words in the middle. By brushing the umap space, the items that contained the brushed words are shown in the table.

By selecting a specific questionnaire domain, the words umap space will change to reflect the words that were used in a certain domain as shown in Figure 6.

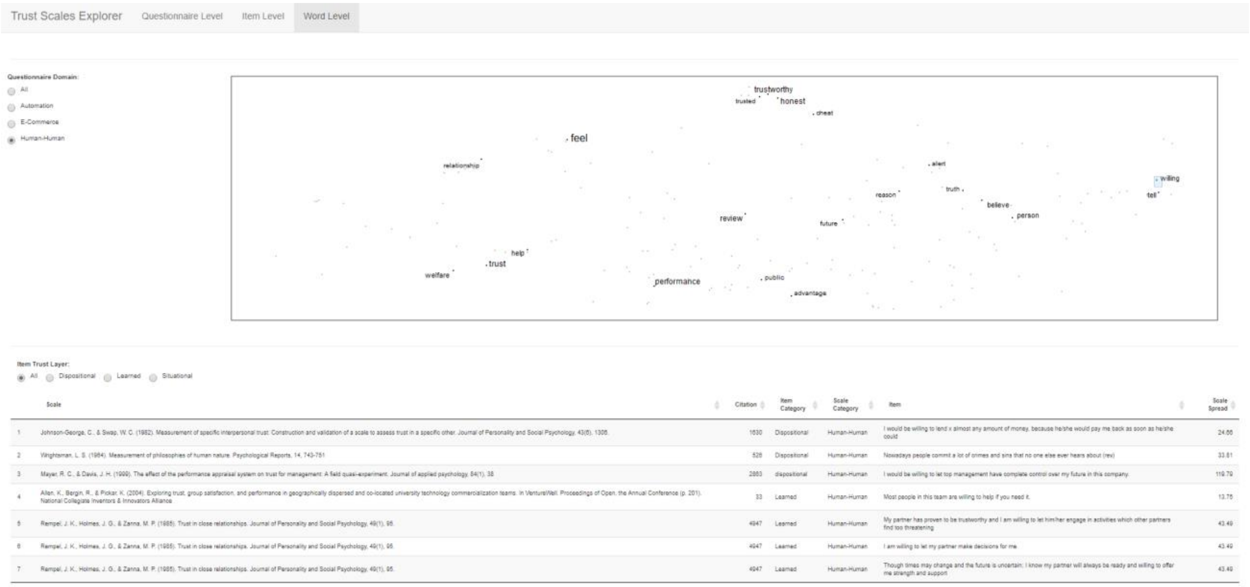

	Scale	Citation	Item Category	Scale Category	Item	Scale Spread
1	Johnson-George, C., & Swap, W. C. (1982). Measurement of specific interpersonal trust: Construction and validation of a scale to assess trust in a specific other. Journal of Personality and Social Psychology, 43(6), 1306.	1630	Dispositional	Human-Human	I would be willing to lend x almost any amount of money, because he/she would pay me back as soon as he/she could	24.66
2	Wrightsman, L. S. (1964). Measurement of philosophies of human nature. Psychological Reports, 14, 743-751	526	Dispositional	Human-Human	Nowadays people commit a lot of crimes and sins that no one else ever hears about (rev)	33.81
3	Mayer, R. C., & Davis, J. H. (1999). The effect of the performance appraisal system on trust for management: A field quasi-experiment. Journal of applied psychology, 84(1), 38	2863	dispositional	Human-Human	I would be willing to let top management have complete control over my future in this company.	119.79
4	Allen, K., Bergin, R., & Pickar, K. (2004). Exploring trust, group satisfaction, and performance in geographically dispersed and co-located university technology commercialization teams. In VentureWell. Proceedings of Open, the Annual Conference (p. 201). National Collegiate Inventors & Innovators Alliance.	33	Learned	Human-Human	Most people in this team are willing to help if you need it.	13.76
5	Rempel, J. K., Holmes, J. G., & Zanna, M. P. (1985). Trust in close relationships. Journal of Personality and Social Psychology, 49(1), 95.	4947	Learned	Human-Human	My partner has proven to be trustworthy and I am willing to let him/her engage in activities which other partners find too threatening	43.49
6	Rempel, J. K., Holmes, J. G., & Zanna, M. P. (1985). Trust in close relationships. Journal of Personality and Social Psychology, 49(1), 95.	4947	Learned	Human-Human	I am willing to let my partner make decisions for me	43.49
7	Rempel, J. K., Holmes, J. G., & Zanna, M. P. (1985). Trust in close relationships. Journal of Personality and Social Psychology, 49(1), 95.	4947	Learned	Human-Human	Though times may change and the future is uncertain, I know my partner will always be ready and willing to offer me strength and support	43.49

Figure 6. Word umap space filtering based on questionnaire domain.
