# Supplementary material for: Measuring trust: a text analysis approach to compare, contrast, and select trust questionnaires
Source: Front Psychol. 2023 Nov 15;14:1192020. doi: 10.3389/fpsyg.2023.1192020 (PMC10684734; doi:10.3389/fpsyg.2023.1192020)
Supplement: Supplementary file 3 [file Data_Sheet_3.PDF]

Appendix C:

List of Manually Removed Words

1. "robot"
2. "gripper"
3. "brand"
4. "technology"
5. "automation"
6. "partner"
7. "system”
8. "user"
9. “systems"
10. "users”
11. "excel"
12. "spreadsheet”
13. "product"
14. "products"
15. "tank"
16. "professional"
17. "professionals"
