# Supplementary material for: Measuring trust: a text analysis approach to compare, contrast, and select trust questionnaires
Source: Front Psychol. 2023 Nov 15;14:1192020. doi: 10.3389/fpsyg.2023.1192020 (PMC10684734; doi:10.3389/fpsyg.2023.1192020)
Supplement: Supplementary file 4 [file Data_Sheet_4.pdf]

Appendix D:
Composition-Based Clustering of Trust Scales

The figure below combines the scales' domain- and layer- composition results. Using cluster analysis, the questionnaires were clustered based on the three layers (situational, dispositional, and learned), and the three domains (automation, e-commerce, and human-human). The dendrogram below. The dendrogram and heatmap displayed below provides a visual representation of the clustering process.

A dendrogram is a hierarchical tree diagram that illustrates the relationships and similarities between clusters. The height or length of the branches in the dendrogram represents the dissimilarity between the clusters. Shorter branches indicate a higher similarity between questionnaires within the same cluster, while longer branches suggest greater dissimilarity between clusters.

In the figure, we can see a dendrogram on the left side, the dendrogram showcases the hierarchical structure of the clustering results, where the questionnaires are grouped together based on their similarities in terms of layer composition (situational, dispositional, and learned) and domain composition (automation, e-commerce, and human-human). Note that this clustering is based on composition similarity and not lexical similarity. On the top, we see a dendrogram based on the layer and domain-composition. The figure shows that most automation questionnaires contain items that assess the learned layer of trust, whereas most human-human questionnaires contain items that assess the dispositional trust layer.

A heat map is a graphical representation of data where values are encoded as colors. In this case, the heat map represents the relationship between the papers and the clustering categories. Each paper is represented by a row, and each clustering category is represented by a column. The heat map uses color gradients to depict the values or relationships between the papers and the clustering categories. High values are represented by lighter colors, while low values are represented by darker colors. For example, Holthausen, Wintersberger, Walker & Riener (2020) is made up 100% of situational items, and almost 100% of automation-related words.

By combining the scales' domain- and layer-composition results and utilizing cluster analysis, we gain insights into the underlying patterns and relationships within the questionnaire data. This analysis facilitates a better understanding of the interplay between different layers and domains, enabling researchers to discern distinct clusters and explore the variations in responses across the identified clusters.

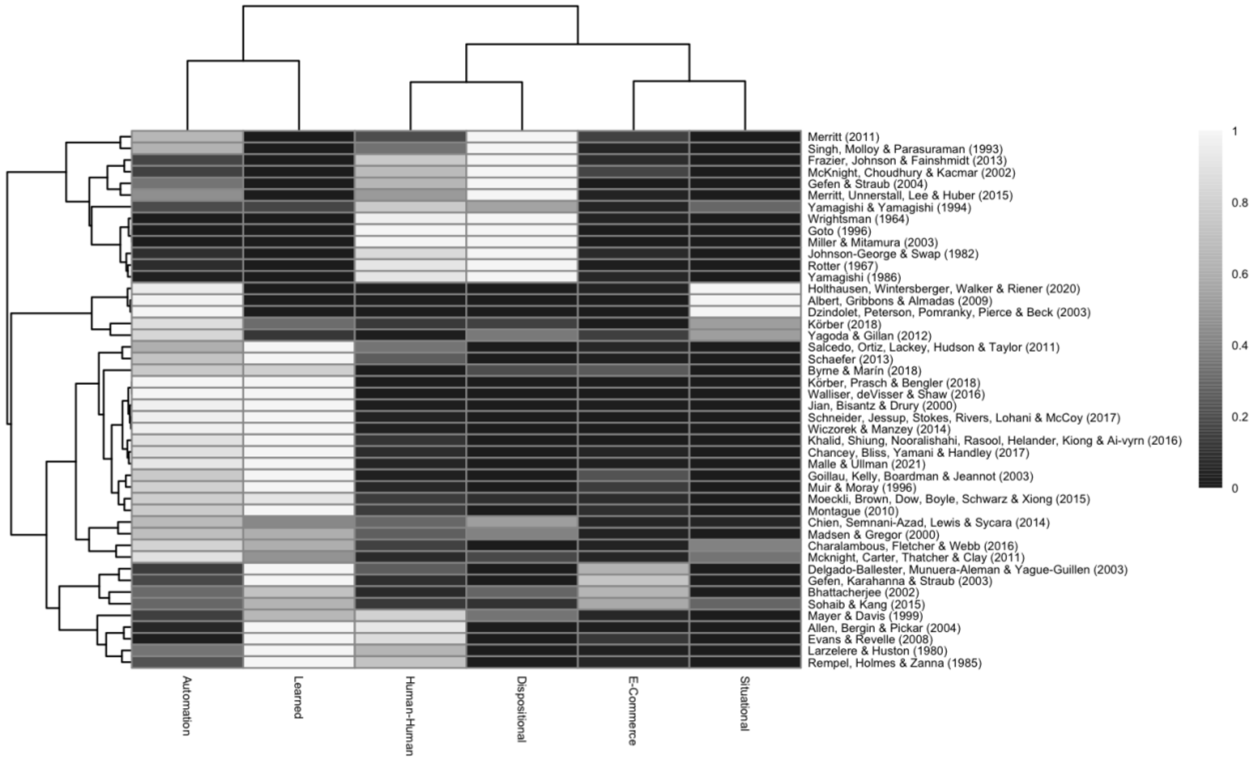

Figure 1. A heatmap and dendrogram of the scales composition.
